# Association of hair glucocorticoid levels with sleep quality indicators: a pilot study in apparently healthy perimenopausal and menopausal women

**DOI:** 10.3389/fendo.2023.1186014

**Published:** 2023-07-17

**Authors:** Eglė Mazgelytė, Agnė Valatkevičiūtė, Jurgita Songailienė, Algirdas Utkus, Neringa Burokienė, Dovilė Karčiauskaitė

**Affiliations:** ^1^ Department of Physiology, Biochemistry, Microbiology and Laboratory Medicine, Institute of Biomedical Sciences, Faculty of Medicine, Vilnius University, Vilnius, Lithuania; ^2^ Department of Human and Medical Genetics, Institute of Biomedical Sciences, Faculty of Medicine, Vilnius University, Vilnius, Lithuania; ^3^ Clinics of Internal Diseases and Family Medicine, Institute of Clinical Medicine, Faculty of Medicine, Vilnius University, Vilnius, Lithuania

**Keywords:** hair glucocorticoids, cortisol, cortisone, sleep quality, Pittsburgh sleep quality index

## Abstract

**Background:**

Poor sleep quality is associated with different physical and mental health diseases. It is proposed that increased hypothalamic-pituitary-adrenal axis activity is a potential contributor affecting sleep pattern and quality. We aimed to analyze the relationship between subjective sleep quality indicators and hair glucocorticoid levels among relatively healthy perimenopausal and postmenopausal women.

**Methods:**

A total of 145 women aged 50–64 y.o. were enrolled in the cross-sectional pilot study. Sleep quality was evaluated using the Pittsburgh Sleep Quality Index, while stress level was measured using the Perceived Stress Scale. Hair cortisol and cortisone levels were determined by ultra-high-performance liquid chromatography-tandem mass spectrometry.

**Results:**

Statistically significant positive relationship was found between hair cortisol concentration and Pittsburgh sleep quality index score. Similarly, statistically significant positive associations were observed between hair total glucocorticoid level and Pittsburgh sleep quality index, sleep disturbance, and Perceived Stress Scale scores. Subjects with prolonged sleep latency had significantly higher hair cortisol and total hair glucocorticoid concentrations compared with individuals whose sleep latency is not disturbed. Additionally, Chi-squared test indicated that lower hair cortisol concentration was significantly related to better sleep efficiency.

**Conclusion:**

Increased hair glucocorticoid (cortisol, cortisone) levels were found to be related with worse sleep quality measured by Pittsburgh sleep quality index score.

## 1 Introduction

Poor sleep behaviour can cause changes in mood and reduce alertness and cognitive performance, and even can be associated with an increased risk of mental health disorders (1). Despite the fact that mental health problems in women have long been underinvestigated, several studies have shown that various social factors such as difficulties in maintaining a balance between work and home, stress or sleep disorders can be associated with an increased risk of developing mental health problems (2, 3). It is suggested that dysregulation of the hypothalamic-pituitary-adrenal (HPA) axis has an impact on the mental health and might be involved in the pathway linking poor sleep quality and mental health disorders (1). Although previous studies showed that different stress-related disorders, including posttraumatic stress disorder (PTSD) (4), depression (5) and adjustment disorder (6) are closely related to elevated levels of glucocorticoid (cortisol, cortisone) levels and aberrant glucocorticoid signalling, there are data that do not fully support this hypothesis. Results of the systematic review conducted by Staufenbiel et al. (7) showed that hair cortisol concentration was increased in patients with major depression while patients with anxiety, including generalized anxiety disorder and panic disorder, had significantly lower hair cortisol level compared with the control group. Furthermore, in patients with bipolar disorders, the cortisol level increased only in individuals with a late age of onset.

Menopause is a period when women are especially prone to develop sleep disorders (8, 9). It is suggested that menopausal women are 3.4 times more likely to report sleep disorders compared with the premenopausal women (8). The cessation of ovarian endocrinological function during menopause results in significantly decreased estrogen and progestogen secretion and these alterations are related to other physical, physiological, and psychological changes that possibly have an impact on sleep quality (9). Although hormonal changes are the primary factors associated with the sleep disturbances, the etiology of sleep disorders in menopausal women are not fully elucidated. It is speculated that vasomotor symptoms, psychosocial factors, or medical conditions such as cardiovascular, neurological, endocrine diseases might be important contributors toward increased risk of sleep disorders in menopausal women (8).

Hypothalamic-pituitary-adrenal (HPA) axis dysfunction is suggested to play a role in the development of sleep disorders, while on the other hand it might be the result of the sleep disorder (10). HPA axis hyperactivity might lead to fragmentation of sleep, decreased slow-wave sleep, shortened sleep time while sleep fragmentation and sleep deprivation can exacerbate HPA axis dysfunction (11). Chronic insomnia without depression occurs with elevated cortisol levels in the evening – this rise in cortisol level may be a primary cause of the sleep disturbance (12). The results of recently published data showed that people who report recurring sleep problems have a higher level of cortisol throughout the day and the total duration of sleep is negatively correlated with circulating cortisol throughout the day. Experimental sleep disturbance in the form of repeated excitations during bedtime induces increased HPA axis activity and results in higher cortisol levels the next day. Additionally, individuals who report fewer sleep disturbances demonstrate a flatter slope of cortisol throughout the day (13, 14).

Previous research mostly relied on cortisol analysis in blood, urine and saliva which accurately reflects changes in cortisol levels at the time of sampling, potentially influenced by daily fluctuations but they are not applicable to measure long-term changes in cortisol concentration (7, 15, 16). Investigation of hormones in serum or plasma samples allows to evaluate the level of cortisol during sleep but this method requires the usage of an intravenous cannula that itself can be a stressor influencing cortisol levels and sleep parameters. Moreover, the sampling of blood at short time intervals generally limits the assessment to unnatural laboratory conditions and to a small number of nights that may not reflect natural changes in sleep pattern. Urine and saliva samples for cortisol and cortisone measurement can easily be collected at home across a longer time period and at specific time points close to the sleep period, thus allowing for the assessment of the consequences of disturbed sleep on the HPA axis activity (17). In the last decade, analysis of cortisol in scalp hair was found to be the most promising methodological approach for assessing long-term HPA axis activity, a biomarker of chronic stress level, and its association with sleep disorders (10). The main advantage of using hair cortisol as a biomarker of chronic HPA axis activity compared with other specimens (i.e., blood serum, saliva, urine) is the fairly predictable growth rate (1 cm/month) that allows retrospective assessment of integrated cortisol secretion during the period from 1 to 6 months (7, 15, 16). Measurement of hair glucocorticoid levels is a compelling instrument to assess long-term cortisol secretion, however, until now, most studies focused on the link between cortisol and chronic stress (18) or psychopathologies, including post-traumatic stress disorder (19), major depression (20) and bipolar disorder (21). The current study aimed to analyze the relationship between subjective sleep quality indicators and hair glucocorticoid levels among relatively healthy perimenopausal and postmenopausal women.

## 2 Materials and methods

### 2.1 Study participants

The minimum sample size calculated using the open-access program G*Power (version 3.1) was 145 individuals (the *a priori* selected research significance level α = 0.05 and the research power - 80%). Thus, the cross-sectional pilot study included 145 consecutively selected apparently healthy females (50–64 y.o.) participating in the national cardiovascular disease prevention program. Subjects were recruited and data were collected between September 2020 and May 2021. The data presented in the current study (sleep quality, stress, anxiety measures and hair glucocorticoid levels) were collected in another study which aimed to evaluate the association between chronic stress biomarkers (hair glucocorticoids) or subjective stress measures (questionnaires) and the prevalence of cardiovascular disease risk factors, as well as 10-year risk of cardiovascular disease. Exclusion criteria were the presence of acute and chronic diseases, including cardiovascular disease, diabetes mellitus and chronic kidney disease, as well as mental diseases and the use of synthetic steroid hormones during the previous 3 months. Participants provided written informed consent before entering the study. The study protocol was approved by the Vilnius Regional Biomedical Research Ethics Committee (No. 2020/8-1254-735). The procedures used in this study adhere to the tenets of the Declaration of Helsinki.

### 2.2 Glucocorticoid analysis in hair samples

Hair glucocorticoid (cortisol and cortisone) levels were determined from the most proximal 3 cm segment of scalp hair collected from the posterior vertex region of the head. A sample preparation procedure was performed using a slightly modified method, as published in our previous paper (22). Chromatographic separation was performed on an ultra-high performance liquid chromatography (UHPLC) system consisting of two Shimadzu LC-30AD binary pumps, a Shimadzu SIL30AC autosampler, and a Shimadzu CTO-20AC column oven (Shimadzu Corporation, Kyoto, Japan). The UHPLC was coupled with a Shimadzu LCMS8060 triple quadrupole tandem mass spectrometer equipped with an electrospray ionization source (Shimadzu Corporation, Kyoto, Japan), which was operated in the positive ionization mode.

Since hair washing frequency is a potential confounder affecting hair glucocorticoid concentrations (23), we divided study participants into three groups based on hair washing frequency (≤1/week, 2–4/week, >5/week) and compared hair glucocorticoid levels between these groups. The results showed no statistically significant differences in hair cortisol (2.62 (2.51) ng/g, 3.69 (6.36) ng/g, 4.06 (16.04) ng/g, p = 0.134, respectively), cortisone (6.76 (5.12) ng/g, 6.61 (4.23) ng/g, 6.07 (5.86) ng/g, p = 0.966, respectively), and total glucocorticoid (12.22 (14.40) ng/g, 10.46 (13.15) ng/g, 8.78 (6.95) ng/g, p = 0.136, respectively) median values among the groups.

### 2.3 Sociodemographic and lifestyle characteristics

Each subject was asked to complete a questionnaire to collect sociodemographic information (i.e., age, marital status, education) and data on lifestyle factors (i.e., physical activity at work and in leisure, smoking status). The physical activity level of the participants was measured using a four-category rating scale. Score 1 of physical activity at work was described as mainly sedentary work, eg, sitting at a desk; Score 2 – necessary to walk during work without lifting heavy items, eg, working in customer service, teaching; Score 3 – intensive walking during work, carrying heavy items, eg, postman’s work, industrial work; Score 4 – intense heavy physical work, lifting heavy items, eg, the work of a nurse. Score 1 of physical activity at leisure time was described as time mainly spent for reading, watching TV or theatrical performances, etc.; Score 2 – going for a walk or cycling at least 4 times per week, light work in the garden, fishing, bowling etc.; Score 3 – at least three times a week time mainly spent running, swimming, playing tennis or some other similar sport; Score 4 – mainly heavy training, running, swimming, skiing, participating regularly in matches a couple of times a week. Subjects were asked to rate their physical activity by selecting one statement from four to best describe their physical activity.

### 2.4 Sleep quality, psychosocial stress and anxiety measures

The Pittsburgh Sleep Quality Index (PSQI) was created to assess sleep quality and disturbances over the past month. The PSQI consists of 19 questions divided into 7 domains: subjective sleep quality, sleep latency, sleep duration, habitual sleep efficiency, sleep disturbances, use of sleeping medications and daytime dysfunction. Each component yields a score ranging from 0 to 3 points, where 0-1 points mean no dysfunction (good sleep quality), and 2-3 points indicate the sleep dysfunction (poor sleep quality). The sleep component scores are summed to yield a total score ranging from 0 to 21 with higher scores indicating worse sleep quality. It is estimated that a global PSQI index score lower than 5 indicates good sleep quality, while a value equal to or higher than 5 means poor sleep quality (24).

Perceived Stress Scale (PSS) is a stress assessment instrument designed to measure the degree to which situations in the life of a subject are considered stressful. Possible total PSS scores can range from 0 to 40 with higher scores indicating a higher perceived stress level. Scores ranging from 0-13 would be considered low stress, 14-26 would be considered moderate stress, and 27-40 would be considered high perceived stress (25). Anxiety level was assessed by the trait scale of the State-Trait Anxiety Inventory (STAI-T). Overall STAI-T scores range from 20 to 80, with higher scores indicating a greater trait anxiety level (26).

### 2.5 Statistical analysis

Statistical analysis was performed using the R software (version 2.4.0). The Shapiro-Wilk test was used to test the normality of the variables. Comparison of median values (IQR) between the two groups was performed using the Mann-Whitney U test. Comparison of median values (IQR) among three or more groups was performed using the Kruskal-Wallis test. Spearman’s correlation coefficients were used to quantify the strength of the correlation between variables. The correlation was considered as a very weak (r ≤ 0.19), weak (0.20 ≤ r ≤ 0.39), moderate (0.40 ≤ r ≤ 0.69), strong (0.70 ≤ r ≤ 0.89) and very strong (0.90 ≤ r ≤ 1.00). To test the hypothesis of independence between categorical variables Pearson’s χ^2^ test or Fisher’s exact test (when expected frequencies lower than 5) was performed. The level of statistical significance was set at 0.05 for the two-tailed test.

## 3 Results

### 3.1 Study group characteristics

Self-reported data showed that most subjects were married (73.79%) and achieved higher education (84.14%). Also, the majority of participants were non-smokers (88.97%) and non-exposed to passive smoking (92.41%). More than half of subjects (59.03%) were inactive during working hours, but most individuals preferred walking and active lifestyle during leisure-time. Also, the majority of women (81.38%) reported being in the menopausal phase, 17.93% were peri-menopausal and one woman (0.69%) did not provide information about the peri/post-menopausal status. Furthermore, none of the study participants were using estrogen therapy. The sociodemographic and lifestyle characteristics of the study sample are shown in **Table 1**.

**Table 1:** Sociodemographic and lifestyle characteristics of the study group.

Variable	Median (IQR) or n (%)
Age (years)	55 (7)
Marital status	
Married	107 (73.79%)
Single	14 (9.60%)
Divorced	19 (13.10%)
Widowed	5 (3.45%)
Education	
Higher (university or non-university)	122 (84.14%)
Upper secondary	19 (13.10%)
Lower secondary	3 (2.07%)
Special middle education	1 (0.69%)
Night job	
Yes	18 (12.41%)
No	127 (87.59%)
Physical activity at work	
Inactive (Score 1)	85 (59.03%)
Walking (Score 2)	45 (31.25%)
Active (Score 3)	12 (8.33%)
Physically hard work (Score 4)	2 (1.39%)
Leisure-time physical activity	
Inactive (Score 1)	44 (30.56%)
Walking (Score 2)	91 (63.19%)
Active (Score 3)	9 (6.25%)
Smoking status	
Yes (everyday)	11 (7.59%)
Yes (sometimes)	5 (3.45%)
No	129 (88.97%)
Passive smoking	
Never or almost never	134 (92.41%)
< 1 hour/day	7 (4.83%)
≥ 1 hour/day	4 (2.76%)

IQR, interquartile range.

### 3.2 Association between Pittsburgh Sleep Quality Index, Perceived Stress Scale, and Hair Glucocorticoid Levels

The median (IQR) values of hair cortisol, cortisone and total glucocorticoid levels were 3.58 (6.76) ng/g, 6.69 (4.95) ng/g and 10.31 (12.17) ng/g, respectively. The comparison of hair steroid hormone concentrations among groups based on different sleep quality and self-reported stress level is reported in **Table 2**.

**Table 2:** Comparison of hair cortisol, cortisone and total glucocorticoid level median values between the groups based on sleep quality indicators and perceived stress level.

Group	Number of subjects n (%)	Hair cortisol (ng/g) median (IQR)	p-value	Hair cortisone (ng/g) median (IQR)	p-value	Hair glucocorticoid (ng/g) median (IQR)	p-value
Subjective sleep quality^1^							
≤ 1	115 (79.86%)	3.83 (7.07)	0.208	6.69 (5.36)	0.464	11.01 (12.71)	0.187
≥2	29 (20.14%)	2.87 (6.03)		5.69 (4.26)		9.42 (8.37)	
Sleep latency^1^							
≤ 1	95 (65.97%)	3.04 (5.62)	**0.018**	5.90 (4.51)	0.086	9.86 (8.29)	**0.017**
≥2	49 (34.03%)	4.98 (16.42)		7.29 (6.27)		12.39 (26.68)	
Sleep duration^2^							
≤ 1	128 (89.51%)	3.13 (6.53)	0.259	6.58 (5.38)	0.269	10.15 (11.11)	0.227
≥2	15 (10.49%)	4.17 (22.17)		7.44 (3.79)		13.17 (21.93)	
Sleep efficiency^3^							
≤ 1	122 (85.92%)	3.10 (6.90)	0.228	6.44 (5.15)	0.675	10.07 (12.75)	0.374
≥2	20 (14.08%)	4.32 (6.34)		6.89 (4.36)		12.08 (11.49)	
Sleep disturbance^1^							
≤ 1	92 (63.89%)	3.10 (5.64)	0.115	5.95 (3.61)	0.087	10.03 (8.95)	0.082
≥2	52 (36.11%)	4.14 (9.79)		7.51 (9.57)		11.52 (24.56)	
Use of sleep medication^1^							
≤ 1	126 (87.50%)	3.13 (6.85)	0.219	6.58 (5.22)	0.528	10.15 (10.72)	0.403
≥2	18 (12.50%)	3.94 (5.89)		7.07 (3.51)		11.77 (15.36)	
Daytime dysfunction^1^							
≤ 1	115 (79.86%)	3.10 (6.48)	0.208	6.59 (4.11)	0.550	10.11 (10.52)	0.195
≥2	29 (20.14%)	5.42 (9.79)		7.44 (8.01)		12.18 (23.31)	
PSQI^4^							
<5	60 (43.48%)	3.13 (5.29)	0.230	5.91 (3.75)	0.387	9.95 (7.84)	0.259
≥5	78 (56.52%)	3.79 (9.03)		6.87 (6.49)		11.46 (16.73)	
PSS^1^							
0-13	50 (34.97%)	2.99 (6.01)	0.730	5.91 (3.45)	0.590	9.69 (13.59)	0.650
14-26	88 (61.54%)	4.07 (6.98)		6.89 (5.58)		11.61 (11.43)	
27-40	5 (3.50%)	3.28 (0.68)		7.72 (1.81)		11.56 (2.74)	

PSQI, Pittsburgh Sleep Quality Index; PSS, Perceived Stress Scale. Statistically significant results are marked in bold.

^1^Variable has missing data (n=144).

^2^Variable has missing data (n=143).

^3^Variable has missing data (n=142).

^4^Variable has missing data (n=138).

The results showed that subjects with increased sleep latency had significantly higher hair cortisol and total hair glucocorticoid concentrations compared with individuals whose sleep latency is not disturbed. Differences in hair steroid hormone levels among the groups based on the other PSQI components (subjective sleep quality, sleep duration, sleep efficiency, sleep disturbance, use of sleep medication, daytime dysfunction), total PSQI score, and perceived stress level were found to be non-statistically significant.

Correlation analyses revealed a weak but statistically significant positive association between hair cortisol concentration and Pittsburgh sleep quality index score. Similarly, statistically significant positive correlations were found between total glucocorticoid level and sleep disturbance, Pittsburgh sleep quality index, and perceived stress scale scores in the current study (**Table 3**).

**Table 3:** Correlations of hair cortisol, cortisone and total glucocorticoid levels with sleep quality indicators, perceived stress and anxiety level.

Variable	Hair cortisol		Hair cortisone		Hair glucocorticoid	
	r_s_	p-value	r_s_	p-value	r_s_	p-value
Subjective sleep quality	-0.017	0.837	-0.001	0.995	-0.020	0.812
Sleep latency	0.151	0.069	0.069	0.405	0.137	0.101
Sleep duration	0.056	0.509	0.106	0.206	0.075	0.375
Sleep efficiency	0.064	0.446	0.059	0.482	0.065	0.439
Sleep disturbance	0.159	0.057	0.147	0.078	0.169	**0.043**
Use of sleep medication	0.125	0.135	0.050	0.549	0.097	0.246
Daytime dysfunction	0.099	0.235	-0.025	0.769	0.076	0.367
PSQI	0.179	**0.032**	0.112	0.180	0.167	**0.045**
STAI	0.150	0.072	0.063	0.450	0.141	0.091
PSS	0.161	0.053	0.155	0.063	0.179	**0.031**

PSQI, Pittsburgh Sleep Quality Index; STAI, State-Trait Anxiety Inventory; PSS, Perceived Stress Scale; r_s_, Spearman’s correlation coefficient. Statistically significant results are marked in bold.

Since there are no generally accepted reference values of hair glucocorticoid levels, we divided the entire study population into two groups based on the median values of hair cortisol, cortisone and glucocorticoid (the sum of cortisol and cortisone) levels. The results of the chi-squared test indicated that higher hair cortisol and cortisone concentrations were associated with the longer sleep latency indicating worse sleep quality. Also, lower hair cortisol concentration was significantly related to better sleep efficiency (**Table 4**).

**Table 4:** Sleep quality and perceived stress indicators among the subject groups based on hair cortisol, cortisone and total glucocorticoid median values.

Variable	Hair cortisol level		*X* ^2^	p-value	Hair cortisone level		*X* ^2^	p-value	Hair glucocorticoid level		*X* ^2^	p-value
	≤3.58 ng/g	>3.58 ng/g			≤6.69 ng/g	>6.69 ng/g			≤10.31 ng/g	>10.31 ng/g		
Subjective sleep quality												
≤ 1	54 (84.7%)	61 (74%)	1.62	0.203	57 (79.2%)	58 (79.5%)	0.02	0.966	55 (75.3%)	60 (83.3%)	1.41	0.235
≥2	18 (15.3%)	12 (26%)			15 (20.8%)	15 (20.5%)			18 (24.7%)	12 (16.7%)		
Sleep latency												
≤ 1	53 (73.6%)	42 (57.5%)	4.15	**0.042**	53 (73.6%)	42 (57.5%)	4.15	**0.042**	53 (72.6%)	42 (58.3%)	3.27	0.071
≥2	19 (26.4%)	31 (42.5%)			19 (26.4%)	31 (42.5%)			20 (27.4%)	30 (41.7%)		
Sleep duration												
≤ 1	67 (93.1%)	61 (83.6%)	3.16	0.076	65 (90.3%)	63 (86.3%)	0.55	0.457	67 (91.8%)	61 (84.7%)	1.75	0.187
≥2	5 (6.9%)	12 (16.4%)			7 (9.7%)	10 (13.7%)			6 (8.2%)	11 (15.3%)		
Sleep efficiency												
≤ 1	65 (90.3%)	57 (78.1%)	4.04	**0.044**	63 (87.5%)	59 (80.8%)	1.21	0.271	64 (87.7%)	58 (80.6%)	1.38	0.241
≥2	7 (9.7%)	16 (21.9%)			9 (12.5%)	14 (19.2%)			9 (12.3%)	14 (19.4%)		
Sleep disturbance												
≤ 1	48 (66.7%)	44 (60.3%)	0.64	0.424	51 (70.8%)	41 (56.2%)	3.36	0.067	49 (67.1%)	43 (59.7%)	0.86	0.355
≥2	24 (33.3%)	29 (39.7%)			21 (29.2%)	32 (43.8%)			24 (32.9%)	29 (40.3%)		
Use of sleep medication												
≤ 1	66 (91.7%)	60 (82.2%)	2.86	0.091	64 (88.9%)	62 (84.9%)	0.49	0.480	66 (90.4%)	60 (83.3%)	1.59	0.207
≥2	6 (8.3%)	13 (17.8%)			8 (11.1%)	11 (15.1%)			7 (9.6%)	12 (16.7%)		
Daytime dysfunction												
≤ 1	61 (84.7%)	54 (74%)	2.55	0.110	58 (80.6%)	57 (78%)	0.14	0.713	60 (82.2%)	55 (76.4%)	0.74	0.388
≥2	11 (15.3%)	19 (26%)			14 (19.4%)	16 (21.9%)			13 (17.8%)	17 (23.6%)		
PSQI												
<5	31 (45.6%)	29 (41.4%)	0.24	0.622	34 (48.6%)	26 (38.2%)	1.49	0.221	35 (47.9%)	27 (37.5%)	1.62	0.204
≥5	37 (64.4%)	41 (58.6%)			36 (51.4%)	42 (61.8%)			38 (52.1%)	45 (62.5%)		
PSS												
0-13	29 (40.8%)	21 (29.2%)	2.56	0.250	30 (42.3%)	20 (27.8%)	4.44	0.123	31 (42.5%)	21 (29.2%)	2.84	0.223
14-26	39 (54.9%)	49 (68.1%)			40 (56.3%)	48 (66.7%)			40 (54.8%)	48 (66.7%)		
27-40	3 (4.2%)	2 (2.8%)			4 (1.4%)	1 (5.6%)			2 (2.7%)	3 (4.2%)		

PSQI, Pittsburgh Sleep Quality Index; PSS, Perceived Stress Scale. Statistically significant results are marked in bold.

## 4 Discussion

The main finding in the current study is the positive correlation between hair cortisol, as well as the sum of cortisol and cortisone, and the total score of the Pittsburgh Sleep Quality Index. Furthermore, a more detailed analysis of the PSQI components revealed significant associations of higher hair cortisol levels with longer sleep latency and poorer sleep efficiency, while a higher hair cortisone level was related to prolonged sleep latency. The total glucocorticoid level (the sum of cortisol and cortisone) was associated with increased sleep disturbance. Cortisol is converted to biologically inactive cortisone by the enzymatic reaction catalysed by the 11β-hydroxysteroid dehydrogenase type 2 (27). Thus, there is a strong biological rationale to measure both hair cortisol and cortisone, as well as the total level of these steroids because it might better reflect HPA axis activity and chronic stress level (28).

Sleep latency, which is defined as the time period in which the patient attempts to sleep until the patient actually falls asleep, is considered one of the most important parameters in studying sleep quality (29). Tell et al. (30) analysed day-to-day variations in sleep behaviours and cortisol diurnal rhythm in women with a diagnosis of early-stage breast cancer and found that longer sleep latency was a predictor of increased salivary cortisol decline from wake-up to bedtime while higher total PSQI score was related to a flatter cortisol linear slope. The flat slope of the diurnal cortisol is estimated to be an indicator of dysregulation of the HPA axis and is associated with the occurrence of physical and mental health problems (31). Another study examining the link between sleep latency and hair cortisol level focused on the paediatric population and reported no significant relationship between long-term cortisol exposure and objectively measured sleep latency (32). Sleep efficiency is another essential indicator that reflects the percentage of total time in bed spent actually sleeping and poor sleep efficiency is mainly related to prolonged sleep latency (29). Studies investigating the association between sleep efficiency and HPA axis activity showed inconsistent results: Räikkönen et al. (33) reported that 8-year-old children with low sleep efficiency had increased cortisol secretion throughout the day while Maurer et al. (34) and Eythorsdottir et al. (32) showed no association of post-awakening cortisol secretion or hair cortisol level and objectively measured sleep efficiency in school-aged or pre-school children. Similarly, a cross-sectional study involving 335 apparently healthy women (72.2% of participants younger than 42 y. o.) found no relationship between salivary cortisol levels in the morning or at bedtime and total PSQI, as well as seven different PSQI domains (2).

It is evident that the aforementioned studies focused on pediatric and young adult populations. However, it is estimated that after the age of 40 years the amount of slow wave sleep decreases, wakefulness during bedtime increases and these factors contribute to the increased risk of developing chronic insomnia or other sleep disorders (17). The large cross-sectional study using the data from the Whitehall II study examined the link between subjectively evaluated sleep disturbances and salivary cortisol awakening response or the slope of diurnal cortisol secretion in a community-based cohort of more than 2700 middle-aged men and women. The results showed that short sleep duration and increased sleep disturbances are related to a shallow diurnal cortisol slope with significantly raised cortisol secretion in the evening (35). Since most studies evaluated HPA axis activity by measuring salivary cortisol level, it is necessary to investigate long-term alterations in basal HPA axis activity and its association with the sleep quality indicators. A recently published paper demonstrated that hair cortisol concentration had a mediating effect between shift work and sleep disorders which were diagnosed if the total score of PSQI was higher than 7 (36). Similarly, Wang et al. reported that hair cortisol concentration was a mediator of the association between work burnout and insomnia (37). The results of our study support the hypothesis that poor sleep quality is related to the increased HPA axis activity and the statistically significant association of hair cortisone level with sleep latency emphasizes the importance of cortisone, the inactive form of cortisol, analysis in order to gain the more precise information about the HPA axis activity.

We also found very weak but statistically significant positive correlation between Perceived stress scale scores and total glucocorticoid level. However, no statistically significant differences of hair cortisol, cortisone and total glucocorticoid median values were observed among PSS groups (low, moderate, high stress level). These findings are consistent with the previous research. Olstad et al. (38) reported no statistically significant association between PSS scores and hair cortisol concentration in women living in socioeconomically disadvantaged neighborhoods. Similarly, O’Brien et al. (39) also found no significant relationship between personal stress evaluated using PSS and hair cortisol concentration in a diverse population of 135 adults.

The study has several limitations that need to be addressed in future research. First, the cross-sectional study design does not provide information about causality in the relationship between sleep quality indicators and hair glucocorticoid levels. However, it is suggested that there is a reciprocal relationship because experimental data showed that sleep deprivation stimulated HPA axis while increased glucocorticoid concentration results in nocturnal awakenings and disturbed sleep (33). Second, there is no standardized method for the measurement of hair glucocorticoid levels and the reference values of hair cortisol and cortisone levels are not available. Third, we evaluated individuals’ sleep quality using PSQI but for the more comprehensive information about the sleep quality objective methods such as polysomnographic measures can be used in future studies. Furthermore, the study population consisted of apparently healthy middle-aged women participating in the national cardiovascular disease prevention program. Thus, our study group might not represent the general female population and the findings of the study should be confirmed in a more heterogeneous study group. Finally, it should be noted that in the context of sleep quality and its associations with hair glucocorticoid levels, which are highly variable among individual subjects, our sample size is insufficient as a *post hoc* analysis of the statistical power showed that our research power was 70%. The results of this pilot study should be confirmed and extended by conducting a study with larger sample size including peri/post-menopausal women with objectively evaluated good and poor sleep quality, as well as more detailed information about the frequency and severity of vasomotor symptoms among study participants.

## 5 Conclusions

Higher hair glucocorticoid (cortisol, cortisone) levels were found to be related with the poor subjectively perceived sleep quality as elevated levels of glucocorticoids were associated with the higher total score of Pittsburgh Sleep Quality Index and its components including sleep latency, sleep efficiency and sleep disturbance. These results indicate that regardless of the direction of the association increased HPA axis activity and reduced sleep quality are highly interconnected.

## Data availability statement

The raw data supporting the conclusions of this article will be made available by the authors, without undue reservation.

## Ethics statement

The studies involving human participants were reviewed and approved by Vilnius Regional Biomedical Research Ethics Committee (No. 2020/8-1254-735). The patients/participants provided their written informed consent to participate in this study.

## Author contributions

EM and AV contributed to writing the original draft. DK, NB, JS and AU contributed to review and editing the draft. EM, AV, NB and DK contributed to the formal analysis and interpretation of the results. EM and DK contributed to study design and conceptualization. All authors contributed to the article and approved the submitted version.
